# P-383. Reasons for discontinuation of CAB+RPV LA for HIV treatment in the OPERA Cohort: A CHORUS survey of healthcare providers

**DOI:** 10.1093/ofid/ofaf695.601

**Published:** 2026-01-11

**Authors:** Ricky K Hsu, Michael D Osterman, Jennifer S Fusco, Rachel Palmieri Weber, Michael G Sension, Michael B Wohlfeiler, Quateka Cochran, Gayathri Sridhar, Vani Vannappagari, Kimberley Brown, Jean A van Wyk, Gregory P Fusco

**Affiliations:** AIDS Healthcare Foundation/ NYU School of Medicine, New York, NY; Epividian, Inc., Raleigh, North Carolina; Epividian, Inc., Raleigh, North Carolina; Epividian, Inc., Raleigh, North Carolina; can community health, Miami Beach, FL; AIDS Healthcare Foundation, Miami, Florida; AIDS Healthcare Foundation, Miami, Florida; ViiV Healthcare, Fairfax, Virginia; ViiV Healthcare, Fairfax, Virginia; ViiV Healthcare, Fairfax, Virginia; ViiV Healthcare, Brentford, UK, Brentford, England, United Kingdom; Epividian, Inc., Raleigh, North Carolina

## Abstract

**Background:**

Recent drug development has focused on long-acting (LA) formulations of antiretrovirals for the treatment of HIV. Cabotegravir + rilpivirine LA (CAB+RPV LA) is the only complete LA antiretroviral therapy (ART) regimen approved for the treatment of HIV-1 in the US with injections every month or every two months. Despite its increasing use, reasons for discontinuation of injections have not been routinely investigated. This study aims to describe reasons for discontinuation of CAB+RPV LA in routine clinical care.

**Figure:**
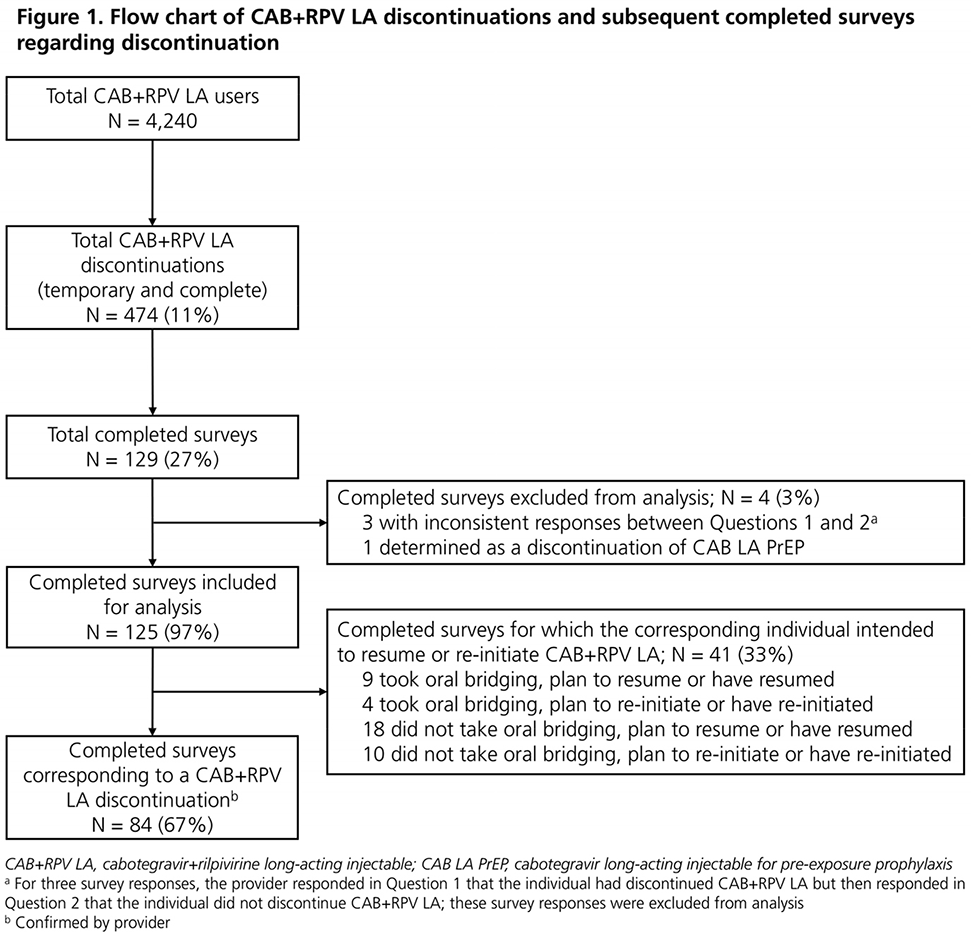

**Figure:**
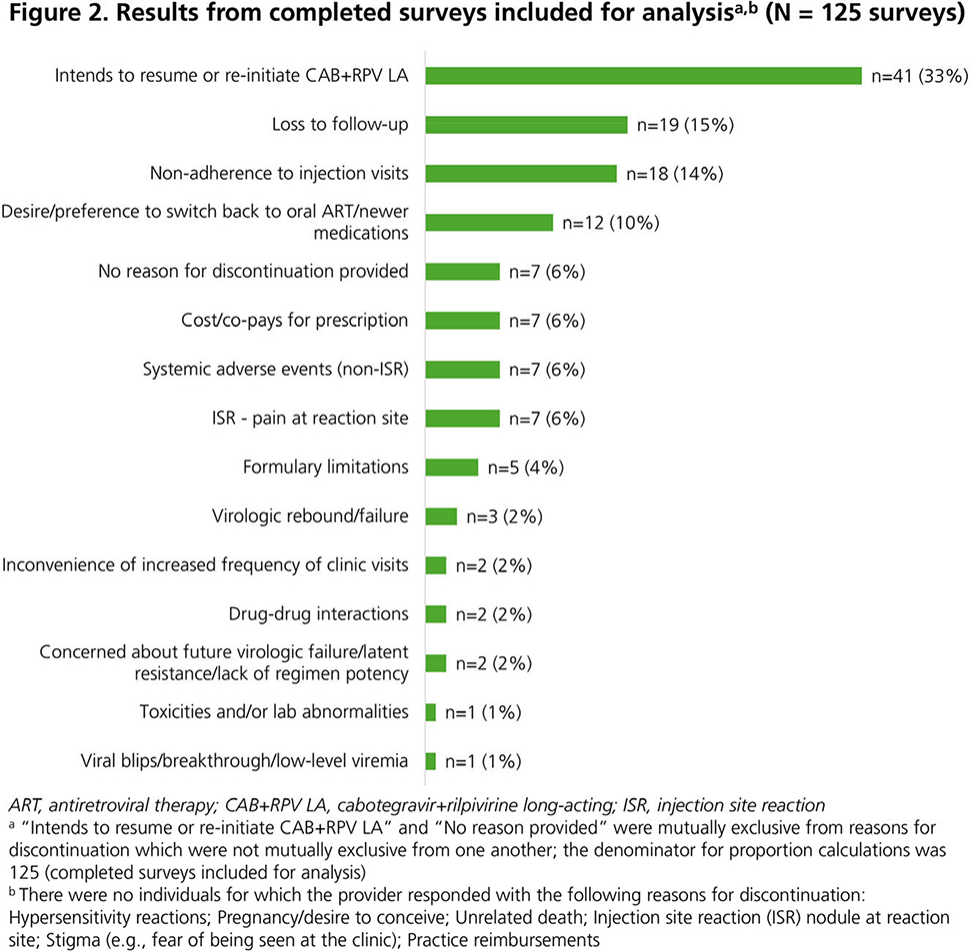

**Methods:**

Healthcare providers (HCPs) that used the CHORUS mobile application between 27JUN2024 and 21JAN2025 were eligible for participation. HCPs were invited to complete a two-question survey for each individual in their care who had either (a) ≥ 61 days since their first CAB+RPV LA injection without another injection or (b) ≥ 61 or ≥ 91 days without an injection after initiating monthly or every other month injections, respectively. The HCP could select reasons for discontinuation in Question 2 after confirming discontinuation in Question 1.

**Results:**

A total of 129 surveys were completed; 125 were included for analysis and 84 corresponded to individuals who discontinued CAB+RPV LA (Fig 1). Forty-one (33%) surveys indicated that the individual intended to restart injections (Fig 2). Seven (6%) surveys provided no reason for discontinuation. Of the remaining 77 surveys, eight included multiple reasons for discontinuation. The most common were loss to follow-up (15%), non-adherence to injection visits (14%), and desire/preference to switch back to oral ART/newer medications (10%). Injection site reactions (ISRs; 6%), drug-drug interactions (2%), and other systemic adverse events (AEs; 6%) were infrequently selected. Virologic rebound/failure (2%) and resistance/potency concerns (2%) were rare (Fig 2).

**Conclusion:**

One-third of providers indicated that the individual planned to restart CAB+RPV LA injections; among the remaining individuals, loss to follow-up or non-adherence to the schedule were the most common reasons for discontinuation whereas concerns about ISRs, AEs, and effectiveness were uncommon. These findings provide insight from HCPs regarding reasons individuals may choose to discontinue CAB+RPV LA for HIV treatment in routine clinical care.

**Disclosures:**

Ricky K. Hsu, MD, Gilead: Advisor/Consultant|Gilead: Grant/Research Support|Gilead: Honoraria|Merck: Honoraria|Serono: Advisor/Consultant|Serono: Honoraria|ViiV: Advisor/Consultant|ViiV: Grant/Research Support|ViiV: Honoraria Michael D. Osterman, PhD, Gilead Sciences: Grant/Research Support|Merck & Co.: Grant/Research Support|Theratechnologies, Inc.: Grant/Research Support|ViiV Healthcare: Grant/Research Support Jennifer S. Fusco, BS, Gilead Sciences: Grant/Research Support|Merck & Co.: Grant/Research Support|Theratechnologies Inc: Grant/Research Support|ViiV Healthcare: Grant/Research Support Rachel Palmieri Weber, PhD, Gilead Sciences: Grant/Research Support|Merck & Co.: Grant/Research Support|Theratechnologies: Grant/Research Support|ViiV Healthcare: Grant/Research Support Michael G. Sension, MD, Gilead: Grant/Research Support|Gilead: Honoraria|Viiv: Honoraria Gayathri Sridhar, MBBS, MPH, PhD, GlaxoSmithKline: Stocks/Bonds (Public Company)|ViiV Healthcare: Full Time Employee Vani Vannappagari, MBBS, MPH, PhD, ViiV Healthcare: Full time Employee of ViiV Healthcare and owns GSK stock|ViiV Healthcare: Stocks/Bonds (Public Company) Kimberley Brown, PharmD, ViiV Healthcare: Employee|ViiV Healthcare: Stocks/Bonds (Public Company) Jean A. van Wyk, MBChB, MFPM, ViiV Healthcare: Employee|ViiV Healthcare: Stocks/Bonds (Public Company) Gregory P Fusco, MD, MPH, Gilead: Grant/Research Support|Merck: Grant/Research Support|Theratechnologies: Grant/Research Support|Viiv: Grant/Research Support

